# Two *Drosophila* Neuropeptide Y-like Neurons Define a Reward Module for Transforming Appetitive Odor Representations to Motivation

**DOI:** 10.1038/s41598-018-30113-5

**Published:** 2018-08-03

**Authors:** Yuhan Pu, Yiwen Zhang, Yan Zhang, Ping Shen

**Affiliations:** Department of Cellular Biology and Biomedical and Health Sciences Institute, University of Georgia, 500 D. W. Brooks Drive, Athens, GA 30602 USA

## Abstract

Neuropeptides, many of which are conserved among vertebrate and invertebrate animals, are implicated in the regulation of motivational states that selectively facilitate goal-directed behaviors. After a brief presentation of appetitive odors, *Drosophila* larvae display an impulsive-like feeding activity in readily accessible palatable food. This innate appetitive response may require coordinated signaling activities of dopamine (DA) and neuropeptide F (NPF; a fly homolog of neuropeptide Y). Here we provide anatomical and functional evidence, at single-cell resolution, that two NPF neurons define a reward module in the highest-order brain region for cognitive processing of food-related olfactory representations. First, laser lesioning of these NPF neurons abolished odor induction of appetitive arousal, while their genetic activation mimicked the behavioral effect of appetitive odors. Further, a circuit analysis shows that each of the two NPF neurons relays its signals to a subset of target neurons in the larval hindbrain-like region. Finally, the NPF neurons discriminatively responded to appetitive odor stimuli, and their odor responses were blocked by targeted lesioning of a pair of dopaminergic third-order olfactory neurons that appear to be presynaptic to the NPF neurons. Therefore, the two NPF neurons contribute to appetitive odor induction of impulsive-like feeding by selectively decoding DA-encoded ascending olfactory inputs and relaying NPF-encoded descending motivational outputs for behavioral execution.

## Introduction

Olfaction is an ancient sense that is crucial for foraging and evaluation of food sources. At present, how chemically diverse volatiles from food are perceived and translated to specific appetitive drives remains poorly understood^1–3^. To induce appetite, an olfactory stimulus must be selectively recognized by a foraging animal as a food-related rewarding cue. Further, the brain may also integrate such appetitive odor stimulation with other senses such as taste to synergistically enhance appetite^4–6^. Therefore, investigation of molecular and circuit mechanisms for neural processing of food odor representations should provide novel insights into the inner workings of the brain and its evolution.

*Drosophila* larvae, like mammals, display an aroused appetitive state following appetitive odor stimulation even under well-nourished conditions^7^. Such motivational states can be quantified based on their increased levels of feeding activity in readily accessible palatable food. A more detailed behavioral analysis showed that fly larvae are capable of assessing the potential appetitive value of a food-related odor stimulus based on the chemical property of the stimulus and its intensity^8^. These observations suggest that fly larvae have evolved a complex brain mechanism for cognitive processing of food-related scents.

Drosophila larvae have a highly evolved yet numerically simple nervous system, providing an experimentally amenable model for brain circuit analysis^9,10^. Anatomical and functional analyses have revealed that the olfactory system of larvae is comprised of 21 olfactory receptor neurons unilaterally^10^, each relaying odor stimulation to one of the 21 uniglomerular projection neurons^11^. Two assemblies of four DA neurons, one in brain hemisphere, appear to be postsynaptic to the second-order projection neurons in the brain regions named lateral horns^7^. At present, how such dopaminergic third-order olfactory neurons contribute to a reward circuit for selective recognition of appetitive odor representations by the larval brain remains incompletely understood.

Our recent genetic analysis suggests that two gene activities are central to neural processing of DA-encoded food odor representations^7^. One of them encodes a D1-like DA receptor Dop1R1. The other gene encodes neuropeptide F (NPF), the fly homolog of mammalian neuropeptide Y (NPY)^12^. Like many other neuropeptides that selectively regulate goal-directed behavioral drives, NPF has been implicated in driving reward-seeking behaviors^13–15^. We have also found that Dop1R1 is likely expressed in NPF neurons, and this Dop1R1 activity may mediate a gating mechanism that tunes NPF signaling in response to appetitive odor-evoked DA inputs^8^.

In this work, we attempt to identify specific NPF neurons that may contribute to a higher-order circuit module for cognitive processing of appetitive odor stimuli. We provide evidence that the activity of a pair of NPF neurons, located in the dorsomedial region of the larval brain, is necessary and sufficient to induce appetitive arousal in fed larvae. Each of the two NPF neurons relays its signal to the target neurons in the larval hindbrain-like region. In addition, these two NPF neurons display selective excitatory responses to stimulation by odor vapors at appetitive doses. Finally, the odor responses of the two NPF neurons were blocked by targeted lesioning of a pair of dopaminergic third-order olfactory neurons, and these NPF neurons appear to be postsynaptically connected with such DA neurons in a brain region named the lateral horn. In combination, our findings suggest that the two NPF neurons contribute to appetitive odor induction of impulsive-like feeding by selectively converting DA-encoded ascending olfactory inputs to NPF-encoded descending motivational outputs for behavioral execution.

## Results

### Functional dissection of the NPF system in odor-aroused appetite

**Figure 1:**
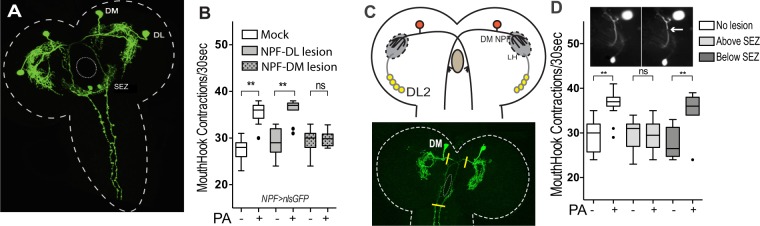
Laser microsurgery analyses of selected NPF neurons in odor-aroused feeding behavior. (**A**) GFP expression driven by *npf-Gal4* in the larval central nervous system. DM: dorsomedial; DL: dorsolateral. SEZ: subesophageal zone. (**B**) The effects of laser lesions in DL- or DM-NPF neurons on the PA-aroused feeding response. (n = 15–17); (**C**) Distribution of the axons and dendrites of the DM-NPF neurons in the larval brain lobes^18^. LH: lateral horns; DL2: the dopaminergic third-order olfactory neuron. The yellow bars in the lower panel indicate severing sites in the two axons by laser microsurgery. (**D**) The effects of laser lesions in the axons of DM-NPF neurons on the PA-aroused feeding response. (n = 10–15); All behavioral assays in this and other figures were quantified under blind conditions. Kruskal-Wallis test was used followed by Dunn’s multiple comparisons test: **P < 0.0001.

In each brain hemisphere of *Drosophila* larvae, an assembly of four dopaminergic third-order olfactory neurons (named DL2-1 to 4)^7^, appears to be postsynaptic to projection neurons. The DL2 neurons have two major roles: integrative processing of olfactory inputs from projection neurons and relaying DA-encoded outputs to Dop1R1 neurons that express NPF^8^. Since both *in situ* RNA and transgenic analyses have mapped NPF expression to two pairs of neurons in the larval brain^12,16^ (also see Fig. 1A), we performed a targeted laser lesion analysis to determine which subset of NPF neurons might be necessary for odor-aroused appetite.

Laser lesions were generated in either the dorsomedial (DM) or dorsolateral (DL) pair of NPF neurons. The feeding activity of fed larvae with lesioned NPF neurons can be quantified by scoring the rate of mouth hook contraction under blind conditions^7,17^. For example, fed larvae pre-stimulated by an appetitive dose of pentyl acetate (PA) vapor, which has a banana-like scent, showed a 20% increase in the mouth-hook contraction rate. The same odor treatment of fed larvae also showed a 50–100% increase of food ingestion in a parallel dyed food assay^7^. We found that fed larvae with lesioned DL-NPF neurons showed normal odor-aroused appetite, but they failed to do so when two dorsomedial (DM) NPF neurons were lesioned instead (Fig. 1B). These results indicate that DL and DM pairs of NPF neurons have distinct functions, and the latter is essential for odor-aroused appetite.

### The NPF neurons transmit descending signals to control feeding behavior

The *npf-lexA* driver, which predominantly labels two DM-NPF neurons, suggests that the axons of DM-NPF neurons may transmit descending NPF signals towards the subesophageal zone (SEZ, Fig. 1C)^18^, where larval feeding control center is located^19^. To test this hypothesis, we performed another laser microsurgery to sever the axons of the DM-NPF neurons (Fig. 1D). When both axons were severed at sites below the SEZ, fed larvae displayed normal PA-aroused feeding response. However, the feeding response was completely abolished when the severing sites were above the SEZ. These results led us to postulate that the DM-NPF neurons may transmit descending NPF outputs that encode motivational signals.

**Figure 2:**
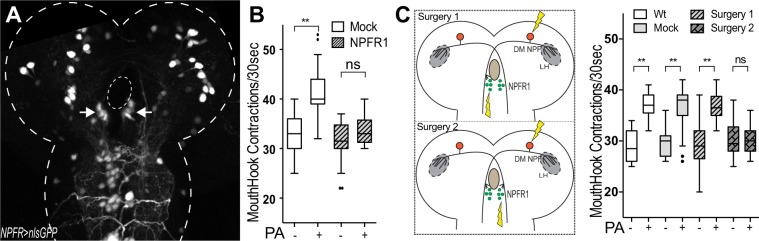
Laser microsurgery analyses of the neuronal pathway comprising DM-NPF and downstream NPFR1-gal4 neurons. (**A**) Expression of nls-GFP in the larval central nervous system driven by *npfr1-Gal4*. The arrowheads indicate two clusters of *npfr1-Gal4* neurons in the hindbrain-like region (SEZ). (**B**) The effects of laser lesions in *npfr1-Gal4* neurons on the PA-aroused feeding response. (n = 12–39); (**C**) A diagram of the two parallel neural pathways comprising DM-NPF and downstream NPFR1-gal4 neurons. Yellow arrows indicate laser lesioning sites. (**D**) The effects of two surgeries on the PA-aroused feeding response. (n = 12–28); Kruskal-Wallis test was used followed by Dunn’s multiple comparisons test: **P < 0.001.

To test this hypothesis, we performed a circuit analysis to determine whether the descending NPF signals act on uncharacterized NPF receptor (NPFR1) neurons in the SEZ. An *npfr*1*-gal4* driver is available that labels two clusters of four SEZ neurons among others (Fig. 2A). These *npfr1-gal4* neurons project their axons to the periphery (Fig. S1). First, we found that laser lesioning of both clusters completely abolished the aroused feeding response in fed larvae, while their baseline feeding activity remained normal (Fig. 2B). Since each DM-NPF neuron projects its axon contralaterally along the midline, we selectively lesioned one DM-NPF neuron in the right hemisphere and one cluster of four *npfr1-gal4* neurons on the contralateral side in the SEZ (Fig. 2C). We found that this microsurgical treatment failed to block the PA-aroused feeding activity. In contrast, when the four *npfr1-gal4* neurons on the ipsilateral side were lesioned instead, the PA-aroused feeding was abolished. These results suggest that the DM-NPF neurons define two parallel neuronal pathways that are functionally autonomous. Consistent with this finding, fed larvae lacking one of the paired clusters of DL2 neurons also displayed normal PA-aroused feeding response^7^.

### Genetic activation of two dorsomedial NPF neurons induces appetitive arousal

**Figure 3:**
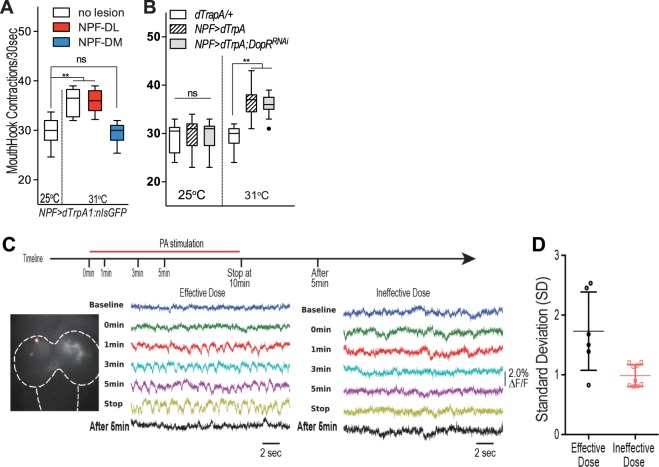
Combined Laser microsurgery, genetic activation and imaging analyses of the DM-NPF neurons. (**A**) Behavioral effects of dTrpA1 activation of one of the two pairs of NPF neurons in the larval brain. The larvae were heat-treated at 31 °C for 15 minutes before the feeding assay in the absence of odor stimulation. One-way ANOVA test was used followed by Tukey’s multiple comparisons test: F (3, 40) = 22.30; (n = 10–12); (**B**) The Behavioral effect of dTrpA1 activation of NPF neurons expressing the RNAi of Dop1R1 (also known as DopR). One-way ANOVA test was used followed by Tukey’s multiple comparisons test: F (5, 98) = 28.77; (n = 17–18); **P < 0.0001. (**C**) ArcLight-based imaging of single dorsomedial NPF neurons (circled) in a preparation of NPF-GAL4/ UAS-*ArcLight* fed larvae. Two sample recordings of normalized membrane activities of dorsomedial NPF neurons from two different preparations: one stimulated by an effective dose of PA and the other stimulated by a higher ineffective dose. (**D**) Standard deviations (SDs) for membrane activity were calculated for both effective (n = 6) and ineffective (n = 9) treatments at 5 minutes after PA stimulation. The effective odor treatment induced significantly stronger excitatory activities of NPF neurons. (t-test, p < 0.01).

We also tested whether activation of the DM-NPF neurons might be sufficient to mimic the appetizing effect of appetitive odor stimulation. A temperature-sensitive TRP family channel (dTrpA1) was used to remotely activate NPF neurons in fed larvae^20^. As a positive control, normal *npf-*GAL4/UAS-*dTrpA1/*UAS-*nls-GFP* fed larvae were heat shocked at 31 °C for 15 min before the feeding assay. These larvae showed increased mouth hook contraction even in the absence of appetitive odor stimulation (Fig. 3A). We then heat shocked the experimental larvae with laser lesions in either DL-NPF or DM-NPF neurons. We found that fed larvae with lesioned DL-NPF neurons showed significantly increased feeding response, while those with lesioned DM-NPF neurons failed to do so. These results suggest that activation of the two DM-NPF neurons is also sufficient to induce appetitive arousal in fed larvae.

A previous genetic analysis showed that heterozygous *Dop1R1*fed larvae failed to show appetitive arousal when stimulated by food odors at normally appetitive doses. Instead, their appetitive arousal requires much stronger odor stimuli that are normally non-appetizing. In addition, functional knockdown of a Dop1R1 activity in *npf-Gal4* neurons also led to similar behavioral phenotypes. These observations suggest that each DM-NPF neuron provides a link between food odor-responsive DL2 neurons and its target neurons in the SEZ, and Dop1R1 may tune the response of NPF neurons to odor-evoked DA signals that are not too strong or too weak^8^. If Dop1R1 acts by gating NPF neuronal responses to DA signals, we would expect that dTrpA1 activation of NPF neurons might lead to bypassing the requirement of Dop1R1 activity to trigger appetitive arousal. Indeed, this is the case. We found that dTrpA1 activation of Dop1R1-deficient NPF neurons was as effective to trigger enhanced feeding activity as its activation of normal NPF neurons (Fig. 3B).

### ArcLight-mediated imaging analysis of odor responses by DM-NPF neurons

We also performed an imaging analysis to directly examine whether DM-NPF neurons display differential cellular responses to appetizing and non-appetizing odor stimuli. We adopted a fluorescent indicator of membrane potential (Arclight) to measure the potential impact of PA stimulation on the membrane potentials of the DM-NPF neurons in a larval preparation^21^. In response to a continuous stream of PA vapor at an effective dose, the DM-NPF neurons showed a gradual increase in excitatory response (Fig. 3C,D). In contrast, in a parallel experiment where a higher ineffective dose of PA vapor was used instead, no excitatory responses were observed, except for a transient depolarization immediately following the odor application. In combination, our findings suggest that the two DM-NPF neurons display differential excitatory responses to odor stimuli of proper intensity, possibly tuned by a Dop1R1-gated intracellular mechanism.

### Anatomical and functional relationships between NPF and DA neurons

**Figure 4:**
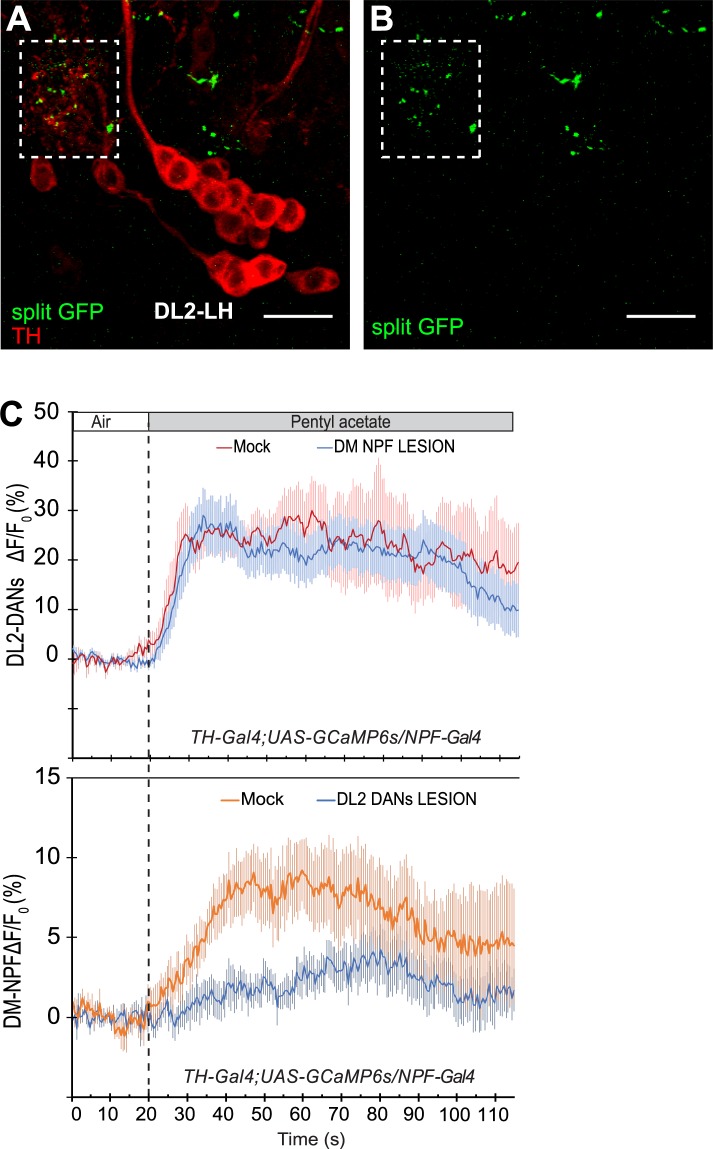
Anatomical and Functional Analyses of Two dorsomedial NPF neurons for Appetitive Arousal. (**A**) Presumptive synaptic connections between NPF and DL2 neurons at the LH (the dotted box) are revealed using a split GFP technique^22^, involving *TH-GAL4*, *NPF-LexA*, *UAS-CD4::spGFP*^*1–10*^ *and LexAop-CD4::spGFP*^*11*^. DL2-LH: four DL2 neurons projecting to the LH. Green: immuno-fluorescence of split GFP; Red: anti-TH. (**B**) The same as the image in panel A, except that anti-TH staining (red) was removed for better viewing of the split GFP signals. Scale bar = 20 μm. (**C**) Calcium imaging analyses of the odor responses of DA and NPF neurons and their functional relationship in odor perception. The DL2 neuronal responses to odor stimuli are independent of DM-NPF neurons, while the DM-NPF neuronal responses, which are slower, require the DL2 neuronal activities. Solid lines and arrow bars show mean ± SE. (DM lesion n = 9, Mock n = 5. DL2 lesion n = 10, Mock n = 8).

The expression pattern of the *npf-*LexA driver also points to the extensive presence of DM-NPF neuronal dendrites in the lateral horn region. In addition, four clustered DL2 neurons are also known to project their axons ipsilaterally to the lateral horn region^7^. To examine how the DM-NPF neurons may receive ascending odor-evoked DA inputs, we first performed an imaging analysis using a split GFP technique^22^. We observed an extensive presence of split-GFP signals in the lateral horn region, suggesting that the DM-NPF neurons likely form synaptic connections with the DL2 neurons (Figs 4A,B and S2).

To provide functional evidence that DM-NPF neurons act downstream from the DL2 neurons, we used a fluorescent Ca^2+^ indicator, GCaMP6 to analyze the functional relationship between the DM-NPF and four DL2 neurons. The excitatory responses of four DL2 neurons and two DM-NPF neurons to PA stimulation were analyzed over a 60-sec period (Fig. 4C). In the presence of the DL2 neurons, the NPF neurons were excited by the PA stimulation, but their odor responses were significantly attenuated when the two clusters of DL2 neurons were lesioned. However, in a reciprocal test, we found that the odor responses of DL2 neurons were similar regardless whether two DM-NPF neurons were lesioned or not. We also noticed that the DL2 neurons responded to the PA stimulation within a few seconds. However, the response of the NPF neurons showed a significant time lag of up to 25 seconds. In combination, our results strongly suggest that DM-NPF neurons act downstream to the food odor-responsive DL2 neurons.

## Discussion

### Role of DM-NPF neurons in cognitive processing of appetitive odor stimuli

**Figure 5:**
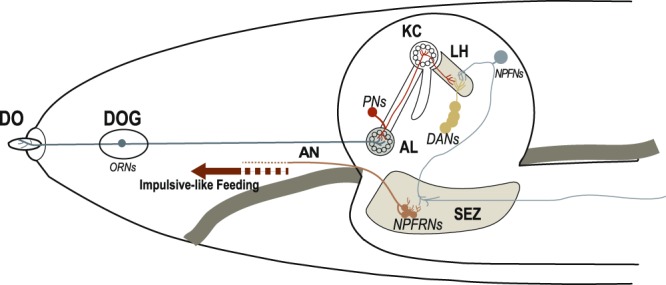
A schematic summary of the proposed roles of DM-NPF and DL2 neurons in food odor perception and feeding motivation. The model depicts a module of a food odor processing circuit in the larval brain hemisphere comprising four DL2 and a downstream DM-NPF. We posit that the DM-NPF neuron defines the highest-order processing module in the circuit. In the lateral horn, the DM-NPF neuron receives ascending presynaptic DA signals encoding appetitive odor representations. Furthermore, the DM-NPF neuron relays descending signals to NPFR1 neurons in the SEZ that control peripheral targets (Fig. S1). Our previous study showed that the appetitive response of fed larvae with reduced Dop1R1 activity in NPF neurons required odor stimuli that are higher than those normally required, as evidenced by a right-shift in the dose-response curve of fed larvae^2^. Together, these findings support the notion that a Dop1R1-mediated intracellular mechanism in the DM-NPF neuron may restrict its responses to selected DA signals, thereby discriminatively assigning appetitive salience to such DA signals. ORNs: olfactory receptor neurons; PNs: projection neurons; DANs: a subset of DA neurons named DL2-1 to 4^7^; NPFN: DM-NPF neuron; NPFRNs: NPFR1 neurons; KC: Kenyon cells; AL: antenna lobes; LH: lateral horns; SEZ: subesophageal zone; AN: antenna nerve; DOG: dorsal organ ganglia; DO: dorsal organ.

We have taken a multifaceted approach to functionally dissect the role of the NPF system in appetitive odor-aroused feeding motivation of *Drosophila* larvae. The anatomical and functional evidence suggest that two DM-NPF neurons, one in each brain hemisphere, define two parallel neuronal pathways that function in a largely autonomous manner (Fig. 5). In each pathway, the DM-NPF neuron defines the highest-order circuit module for food odor processing. In the lateral horn, the DM-NPF neuron receives ascending DA signals from four upstream DL2 neurons. Subsequently, it selectively converts such inputs to descending NPF-encoded motivational outputs, which are relayed to a subset of NPFR1 neurons in larval hindbrain-like region (SEZ) for organizing feeding-related peripheral activities. Through combined use of targeted laser microsurgery and dTrpA1-mediated neuronal activation, we also show that remote activation of two DM-NPF neurons in behaving fed larvae appears to be sufficient to mimic the appetizing effect of food odor stimulation. Therefore, our findings suggest that the DM-NPF neurons define a module of the highest order in a food reward circuit that prepares larvae for reward-driven feeding of palatable food.

### Responses of DM-NPF neurons to appetitive and non-appetitive odor stimuli

One of the key features of the appetitive odor-aroused feeding response by fed larvae is its requirement of optimal levels of odor stimulation; Odor stimuli that are either too strong or too weak are not effective^8^. In heterozygous *Dop1R1* fed larvae, the effective doses of odor vapors required to induce appetitive arousal were much higher, as evidenced by the right-shift in its dose-response curve^8^. Further, fed larvae with a reduced *Dop1R1* activity in NPF neurons also phenocopied heterozygous *Dop1R1* fed larvae. In this work, we have provided cellular evidence that DM-NPF neurons display excitatory responses only to odor stimuli at appetitive doses (Fig. 3). Together, these findings point to the presence of a Dop1R1-mediated gating mechanism that tunes the NPF neuronal response to odor-evoked DA signals, and thereby selectively assigns appetitive significance to DA signals that are otherwise meaningless behaviorally.

### Functional relationship between DL2 and DM-NPF neurons

We have provided several lines of evidence suggesting that in each brain hemisphere, the DM-NPF neuron receives ascending DA signals from an assembly of four dopaminergic DL2 neurons in the lateral horn. First, using an *npf-lexA* driver that predominantly labels the two DM-NPF neurons, we found the dendrites of these neurons were highly enriched in the lateral horn, which is known to mediate innate olfactory behaviors^7^. Second, the four DL2 neurons, which function as third-order olfactory neurons, were previously shown to project their axons exclusively to the lateral horn^7^. Third, the split GFP assay also points to the presence of synaptic connections between the DM-NPF and the upstream DL2 neurons in the lateral horn. Finally, the functional imaging analysis shows that the DM-NPF neuron acts downstream from four DL2 neurons. When stimulated by a stream of appetitive odor vapor, the DL2 neurons responded more rapidly than the NPF neuron, and lesions in the DM-NPF neuron had no effect on the odor response of the DL2 neurons. In contrast, the NPF neuronal response to the same odor stimulus required the presence of the DL2 neurons. In combination, our findings have revealed a previously uncharacterized brain center where a DA/NPF-mediated circuit mechanism underlies cognitive processing of food odors for appetitive motivation.

In summary, we have provided evidence that the activity of a pair of NPY-like neurons defines a reward system in fly larval brain responsible for cognitive processing of food-related olfactory representations. However, when the two DM-NPF neurons were selectively lesioned, the rapid responses of DL2 neurons to a PA stimulus (within 2–5 seconds) remained intact (Fig. 4C). In a previous study, functional knockdown of the NPFR1 activity in the DL2 neurons attenuated their rapid excitation by a transient odor stimulus (e.g., a puff of PA vapor)^7^. Therefore, these findings have raised the possibility that the two DL-NPF neurons, which project their axons ipsilaterally within the brain lobe^18^, may define a separate neural mechanism, and this NPF mechanism may set the basal level of NPF activity in un-stimulated larval brains to facilitate the sensitive detection of distant food sources by foraging larvae.

## Materials and Methods

### Fly Stocks and Larval Growth

All flies are in the w^1118^ background. Larvae were reared at 25 °C, and early third instars (~74 hr after egg laying, AEL) were fed before behavioral experiments as previously described^7^. The transgenic flies including TH-GAL4^23^, UAS-*dTrpA1*^20^,UAS-GCaMP6.0 s (BL42749), UAS-*Arclight* (BL51056), UAS-GPF.nls (BL4776) and *npf-GAL4* were obtained from Bloomington stock center. UAS-*Dop1R1*^*RNAi*^ (V107058) was obtained from the Vienna Drosophila RNAi Center. UAS*-CD4*::*spGFP*^*1–10*^, *LexAop-CD4*::*spGFP*^*11*^ were kindly provided by K. Scott^19^.

### Behavioral Experiments

Quantification of mouth hook contraction rate in liquid food was performed as previously described^24^.A published protocol for fly larvae odor stimulation was used with slight modifications^7^. Briefly, synchronized early third instars, fed on yeast paste, were stimulated for 5 minutes in a sealed 1.5L-chamber that was fumigated with an appetitive dose (5 or 7.5 μl) of pentyl acetate (PA; Sigma-Aldrich, 628-63-7). After rinsing with water, larvae were tested for their feeding responses. Feeding media include agar paste (US Biological, A0940) containing 10% glucose. UAS-*dTrpA*1 was expressed by allowing larvae to feed in pre-warmed yeast paste in a 31 °C incubator for defined periods, followed by rinsing with 31 °C water prior to feeding assays.

### Molecular Cloning and Immunostaining

To construct the npf-LexA driver, a DNA fragment of ~1-kb containing a region spanning from the 5′ regulatory sequence to the beginning of the second axon was amplified by genomic PCR. This fragment was subsequently cloned into the KpnI site in the pBPnlsLexA::GADflUw vector. Forward Primer: cagggagagagaacggagac; Reverse primer: gtgtcacaatgcaattgttcg. Tissue dissection and fixation, antibodies used and dilution conditions were described previously^7^.

### Targeted Laser Lesion

Protocols for calibration of 337 nm nitrogen laser unit and laser lesion experiments have been described^17^.

### GCaMP imaging

The neural tissues of larvae were processed as previously described^7^. A Ca^2+^ indicator, GCaMP6s, was used for imaging odor responses by DA and NPF neurons. The odor delivery system involves a sealed 1.25 L glass chamber fumigated with 7.5 μl PA for 5 minutes. Odor was continuously delivered to larval head region by pumping at a rate of 0.28 L/min. The treated larval preparation was imaged using a Zeiss LSM 510 confocal microscope.

### Arclight imaging and data processing

The method for making larval CNS preparation is the same as previously described for calcium imaging preparation^7^. The preparation was incubated in Drosophila PBS. Effective and ineffective odor vapors were prepared by fumigating a sealed 24 L foam box with 150 or 800 µl PA for 2 hours, respectively. Odor was continuously delivered to larval head region by pumping at a rate of 0.36 L/min.

The protocol for ArcLight Imaging^21^ was followed with minor modifications. Briefly, larval CNS was imaged under 40X water immersion lens using Zeiss Axio Examiner. NeuroCCD-SM camera and Turbo-SM software (RedShirt Imaging) were used for recording and data processing. Images were captured at a frame rate of 100 Hz, and exposure time is 10 ms. 2000 frames were collected for each of the seven 20 s periods.

### Statistical analysis

Statistical analyses for behavioral assays were performed using Kruskal-Wallis followed by Dunn’s multiple comparisons test or one-way ANOVA followed by Tukey’s multiple comparisons.

### Data availability statement

The datasets generated during and/or analysed during the current study are available from the corresponding author on reasonable request.

## Electronic supplementary material

Supplemental information
