# Supplementary material for: Two Drosophila Neuropeptide Y-like Neurons Define a Reward Module for Transforming Appetitive Odor Representations to Motivation
Source: Sci Rep. 2018 Aug 3;8:11658. doi: 10.1038/s41598-018-30113-5 (PMC6076267; doi:10.1038/s41598-018-30113-5)
Supplement: Supplementary file 1 — Supplemental information [file 41598_2018_30113_MOESM1_ESM.pdf]

Supplemental information

Two *Drosophila* Neuropeptide Y-like Neurons Define a Reward Module for Transforming Appetitive Odor Representations to Motivation

Yuhan Pu[1], Yiwen Zhang[1], Yan Zhang and Ping Shen*

Department of Cellular Biology and Biomedical and Health Sciences Institute, University of Georgia, 500 D. W. Brooks Drive, Athens, GA 30602, USA

[1] Co-first authors

* Correspondence should be addressed to P.S. (pshen@uga.edu)

Figure S1 Anatomical analysis of NPFR1-Gal4 neurons in the SEZ
A pair of four NPFR1-Gal4 neurons, labeled by mCD8::GFP, is present in the SEZ. The inset shows a magnified view of their axons exiting from the antenna nerve.

Figure S2 Images of control larvae from the split GFP analysis
A) the image of the brain lobe from *TH-Gal4 /UAS-CD4::spGFP*$^{1-10}$ *; LexAop-CD4::spGFP*^{11} larvae; **B**) the image of the brain lobe from *NPF-Gal4 /UAS-CD4::spGFP*$^{1-10}$ *; LexAop-CD4::spGFP*^{11}larvae. No split GFP signals were detected in the control brain tissues. N = 6-8.

FigS1

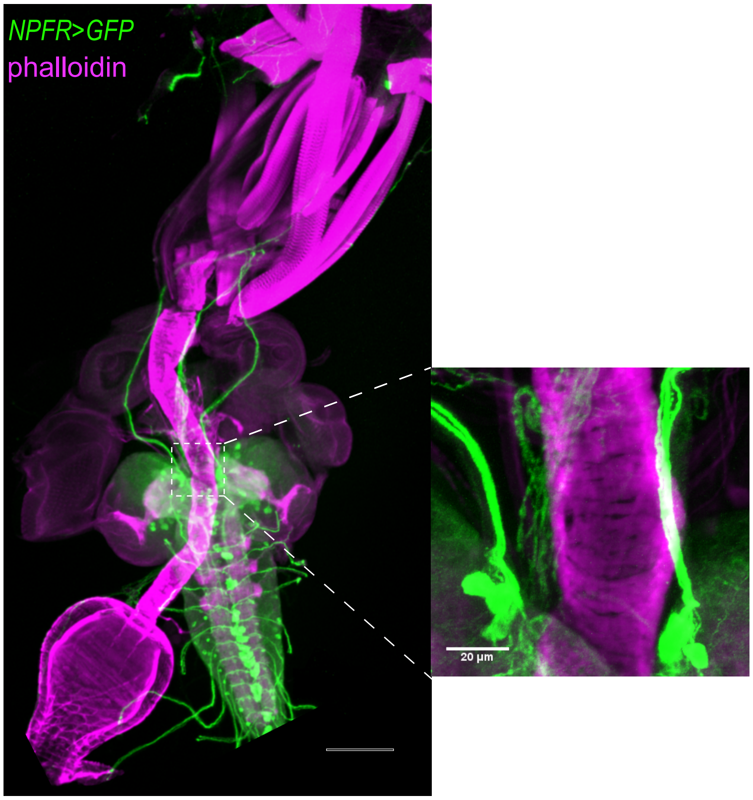

FigS2

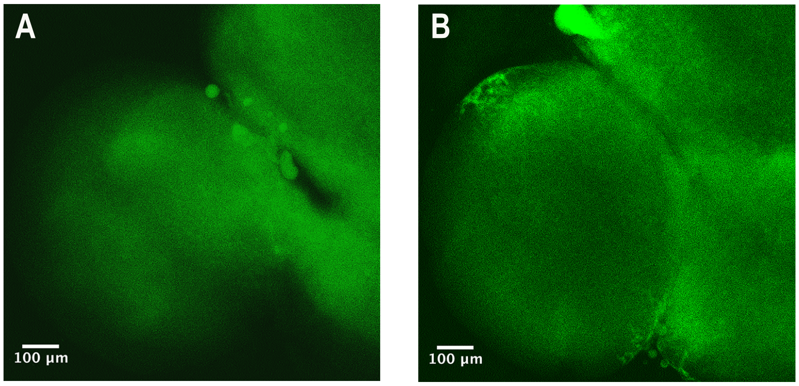

TH-Gal4 /UAS-CD4::spGFP[1-10] *; LexAop-CD4::spGFP*[11]

NPF-LexA/UAS-CD4::spGFP[1-10] *; LexAop-CD4::spGFP*[11]
